# Assessment of the Immunoprotective Efficacy of Recombinant 14-3-3 Protein and Dense Granule Protein 10 (GRA10) as Candidate Antigens for Rabbit Vaccines against *Eimeria intestinalis*

**DOI:** 10.3390/ijms241914418

**Published:** 2023-09-22

**Authors:** Changming Xiong, Wei He, Jie Xiao, Ge Hao, Jiayan Pu, Hao Chen, Liwen Xu, Yuhua Zhu, Guangyou Yang

**Affiliations:** Department of Parasitology, College of Veterinary, Sichuan Agricultural University, Chengdu 611130, China; xiongchangming@stu.sicau.edu.cn (C.X.); 2021303079@stu.sicau.edu.cn (W.H.); 2018203021@stu.sicau.edu.cn (J.X.); haoge@stu.sicau.edu.cn (G.H.); pujiayan@stu.sicau.edu.cn (J.P.); 2020303084@stu.sicau.edu.cn (H.C.); xuliwen@stu.sicau.edu.cn (L.X.); zhuyuhua@stu.sicau.edu.cn (Y.Z.)

**Keywords:** *Eimeria intestinalis*, rabbit, recombinant subunit vaccine, 14-3-3 protein gene, dense granulocyte protein gene

## Abstract

*Eimeria intestinalis* infects rabbits, causing severe intestinal coccidiosis. Prolonged anticoccidial drug use might lead to coccidia resistance and drug residues in food. Thus, vaccines are required to control rabbit coccidiosis. In this study, recombinant *E. intestinalis* 14-3-3 and GRA10 proteins (r*Ei*-14-3-3 and r*Ei*-GRA10) were obtained via prokaryotic expression and used as recombinant subunit vaccines. Fifty 30-day-old rabbits were randomly grouped as follows: PBS-uninfected group, PBS-infected group, Trx-His-S control group, and r*Ei*-14-3-3 and r*Ei*-GRA10 immunized groups. The rabbits were subcutaneously immunized twice at 2-week intervals, challenged with 7 × 10^4^ sporulated oocysts, and sacrificed 14 days later. The protective effects were assessed via clinical signs, relative weight gain, oocyst reduction, mean intestinal lesion score, ACI (anticoccidial index), cytokine, and specific antibody levels in sera. The r*Ei*-14-3-3 and r*Ei*-GRA10 groups had higher relative weight gain rates of 81.94% and 73.61% (*p* < 0.05), and higher oocyst reduction rates of 86.13% and 84.87% (*p* < 0.05), respectively. The two immunized groups had fewer intestinal lesions (*p* < 0.05) and higher IgG levels (*p* < 0.05). Higher levels of IL-2, IL-4, and IFN-γ cytokines in the r*Ei*-14-3-3 group (*p* < 0.05) and a higher level of IFN-γ in the r*Ei*-GRA10 group (*p* < 0.05) were observed. The ACI values of the r*Ei*-14-3-3 and r*Ei*-GRA10 groups were 168.24 and 159.91, with good and moderate protective effects, respectively. Both r*Ei*-14-3-3 and r*Ei*-GRA10 induced humoral immunity in the rabbits. In addition, r*Ei*-14-3-3 induced Th1- and Th2-type immune responses. Both recombinant proteins were protective against *E. intestinalis* infection in rabbits, with r*Ei*-14-3-3 showing a better protective effect.

## 1. Introduction

Rabbit coccidiosis, one of the main diseases affecting rabbit farming, is caused by parasites of the genus *Eimeria*, which parasitize rabbit intestinal or bile duct epithelial cells [1]. Young rabbits aged 1 to 3 months are most susceptible and show a high mortality [2,3]. Among the 11 valid species of rabbit coccidia [3,4], *Eimeria intestinalis* is one of the most pathogenic species and mainly invades the jejunum and ileum [5,6,7]. It causes the most harm to the jejunum, causing the death of massive goblet cells and a reduced secretion of protective glycoproteins, which, in turn leads, to the disruption of the intestinal structure and homeostasis, and dysbacteriosis. The rabbit is then susceptible to secondary infection with enteric pathogens, such as *Escherichia coli*, which aggravates enteritis [8]. While the jejunum is the longest part of the small intestine and has the most important task of absorbing nutrients, its destruction will lead to weight loss, malnutrition, and diarrhea in sick rabbits, and even death in severe cases [9].

The prolonged use of anticoccidial drugs, such as robenidine, diclazuril, and toltrazuril, might lead to problems such as coccidial resistance and drug residues in food [10]. Therefore, it is more promising to prevent and control rabbit coccidiosis via vaccination. Recent research on rabbit coccidia vaccines has focused on a precocious line of live vaccines, such as *E. media* [11], *E. intestinalis* [12], and *E. magna* [13], which have shown good anticoccidial effects. However, live vaccines carry the risk of virulence reversion and are expensive to produce [14]. Recombinant subunit vaccines have the advantages of safety, stability, and lower production costs, and are therefore more promising for large-scale production [15]. The identification and proper selection of protective antigens are crucial for recombinant subunit vaccine development [15,16]. To date, there has been only one report on recombinant subunit vaccines for *E. intestinalis* [17].

The 14-3-3 protein is widely present in eukaryotes and is crucial for the invasion and growth process of Apicomplexan parasites [18]. According to reports, the *Toxoplasma gondii* 14-3-3 gene (*Tg-14-3-3*) might contribute to the transmission of *T. gondii* to host cells, and *T. gondii* cannot survive after its knockout [19]. *E. tenella* 14-3-3 (*Et*-14-3-3) can affect the invasion process and life cycle by interacting with telomerase and calcium-dependent protein kinase 4 (CDPK4) [20,21]. The dense granule (DG) organelle of the Apicomplexan parasite secretes a family of dense granule proteins (GRAs) that are essential for the development of Apicomplexan parasites within the host cell [22]. *Toxoplasma gondii* begins to secrete GRAs after the invasion of host cells [23], most of which are either solubilized in the parasitophorous vacuole (PV) to maintain a normal PV function, are bound to the PV membrane (PVM) as protective antigens against the attack of lysosomes in host cells [24,25], or are transported to the cytoplasm and nucleus of the host cell to participate in the regulation of important life activities, such as the immune response of host cells, nutrient transport, and cell cycles [22,26]. They may both be potential drug targets or vaccine-candidate antigens that are capable of blocking coccidia’s life cycle [20,26].

In this study, the *Ei*-14-3-3 and *Ei*-GRA10 genes were screened for prokaryotic expression based on the transcriptome data of *E. intestinalis* generated in our laboratory. Then, the two recombinant proteins were used as recombinant subunit vaccines, and their immunoprotective effects were evaluated using animal experiments to supply a candidate antigen reference for the development of recombinant subunit vaccines against rabbit coccidia.

## 2. Results

### 2.1. Gene Cloning and Bioinformatic Analysis

We successfully amplified the sequences for *Ei-*14-3-3 (GenBank accession number: OQ428177) and *Ei-*GRA10 (GenBank accession number: OQ428178). The sequence analysis revealed that the ORF of *Ei*-14-3-3 was 816 bp, encoding a protein of 271 amino acids, with a theoretical relative molecular weight of 31.13 KDa and a theoretical isoelectric point (PI) of 4.82. The ORF of *Ei*-GRA10 was 633 bp, encoding a protein of 210 amino acids, with a theoretical relative molecular weight of 20.67 kDa and a theoretical isoelectric point (PI) of 6.94. *Ei*-14-3-3 and *Ei*-GRA10 did not have a signal peptide or a transmembrane region and were predicted to have 14 and 7 B-cell antigen epitopes, respectively.

**Figure 1:**
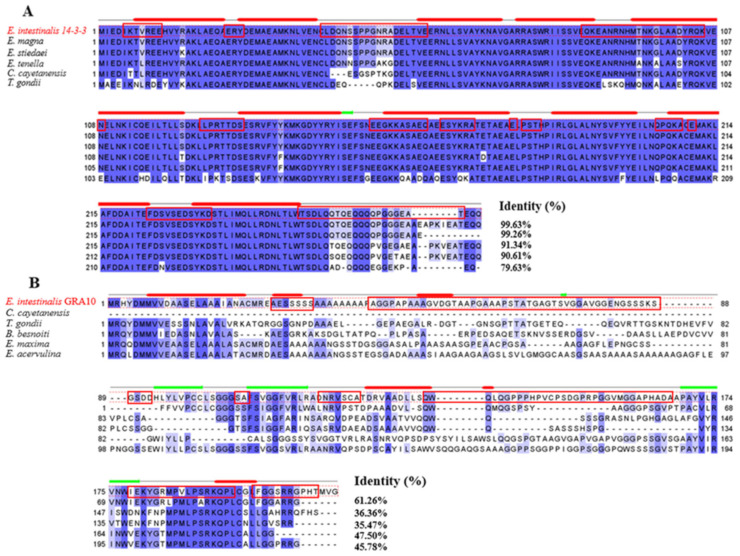
Multiple sequence alignments of 14-3-3 (**A**) and GRA10 (**B**) from different parasites. (**A**) Multiple alignments of *E. intestinalis* 14-3-3 with 14-3-3 proteins from other parasites: *Eimeria magna* (GenBank: OQ632930), *Eimeria stiedaei* (GenBank: UDM59931.1), *Eimeria tenella* (GenBank: XP_013229533.1), *Cyclospora cayetanensis* (GenBank: XP_026194077.1), and *Toxoplasma gondii* (GenBank: XP_002365409.1). (**B**) Multiple alignments of *E. intestinalis* GRA10 with GRA10 proteins from other parasites: *Cyclospora cayetanensis* (GenBank: OEH80410.1), *Toxoplasma gondii* (GenBank: KFG61447.1), *Besnoitia besnoiti* (GenBank: XP_029219573.1), *Eimeria maxima* (GenBank: XP_013333610.1), and *Eimeria acervulina* (GenBank: XP_013251490.1). Blue shading indicates conserved amino acid residues. Red boxes indicate B-cell epitopes.

The multiple sequence comparisons of *Ei*-14-3-3 and *Ei*-GRA10 with the homologues showed that *Ei*-14-3-3 was highly conserved, sharing over 99% of amino acid sequence identity with the 14-3-3 proteins from *E. stiedae* and *E. magna*, while the *Ei*-GRA10 gene was relatively less conserved (Figure 1). The blue color in Figure 1 indicates conserved residues, and the percentage of similarity between two amino acid sequences is marked at the end.

### 2.2. Expression of rEi-14-3-3 and rEi-GRA10

**Figure 2:**
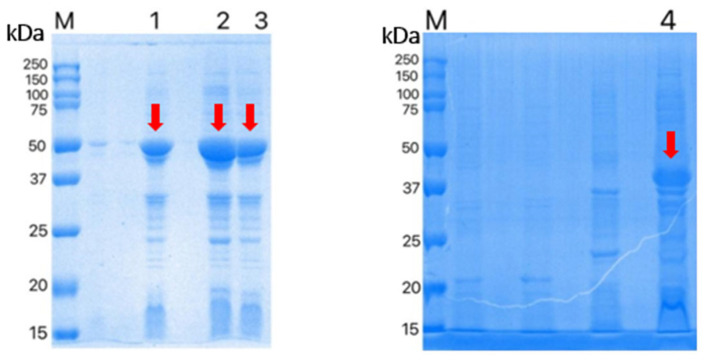
Expression of r*Ei-*14-3-3 and r*Ei-*GRA10 in *E. coli*. M: protein markers; lanes 1–3: r*Ei-*14-3-3; lane 4: r*Ei-*GRA10. Red arrows are used to indicate bands.

The two recombinant proteins were significantly expressed after 1.0 mmol/L of IPTG induction at 37 °C for 8 h. The SDS-PAGE gel electrophoresis results show that the sizes of r*Ei*-14-3-3 and r*Ei*-GRA10 were around 50 KDa and 40 KDa, respectively (Figure 2). The molecular sizes of the two proteins were similar to the theoretical results, excluding the 20 kDa tagged fusion peptide encoded by vector pET-32a, and indicating the successful expression of the two recombinant proteins. The SDS-PAGE results show a good purification of the two recombinant proteins via Ni^2+^-affinity chromatography.

### 2.3. Western Blot Analyses

**Figure 3:**
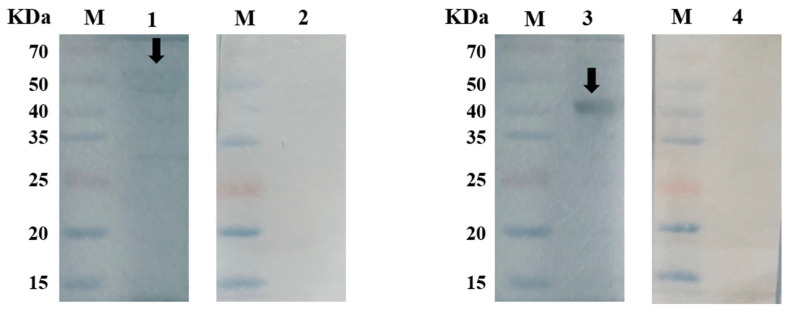
Western blot results. M: protein marker lane 1: r*Ei-*14-3-3 incubated with positive sera; lane 3: r*Ei*-GRA10 incubated with positive sera; lane 2: r*Ei-*14-3-3 incubated with negative sera; lane 4: r*Ei*-GRA10 incubated with negative sera. Black arrows are used to indicate bands.

Specific bands were observed on the PVDF membranes formed by the reaction of r*Ei*-14-3-3 and r*Ei*-GRA10 with anti-*E. intestinal*-positive rabbit sera, respectively, while no specific bands were seen after an incubation with negative sera (sera from one-month-old coccidia-free rabbits), proving the high reactivity of both r*Ei-*14-3-3 and r*Ei-*GRA10 (Figure 3).

### 2.4. Evaluation of the Protective Effect of rEi-14-3-3 and rEi-GRA10

After the first and second vaccinations, neither immunized group experienced any notable adverse responses, and there was no discernible difference among all the groups (*p* > 0.05) in terms of the average weight gain (ΔW1), indicating that the recombinant proteins had a good safety profile. On days 10 and 11 after the challenge, more than six rabbits in both the PBS-infected and Trx-His-S-infected groups (controls were immunized with PBS and pET-32a empty proteins, respectively) showed symptoms of diarrhea and mental depression, whereas only two experimental rabbits in the immunized groups showed symptoms of mild diarrhea. No mortality was observed in any of the groups after the challenge.

**Figure 4:**
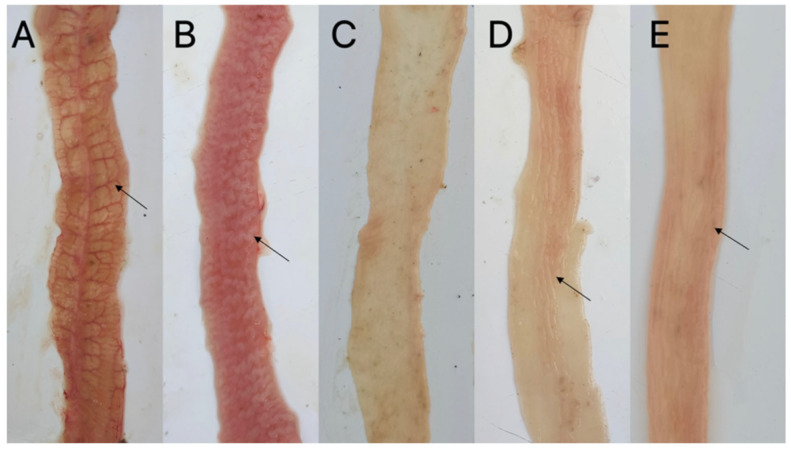
Lesions in the intestine of each group after *E. intestinalis* challenge. (**A**) PBS-infected group: intestinal tract bleeding; (**B**) Trx-His-S-infected group: intestinal wall thickening; (**C**) uninfected group; (**D**) r*Ei*-14-3-3 immunized group: slight intestinal wall thickening; (**E**) r*Ei*-GRA10 immunized group: slight intestinal wall thickening. Black arrow: intestinal wall bleeding or thickening.

No intestinal lesions were seen in the uninfected group, while the PBS-infected and Trx-His-S-infected groups showed the typical pathological changes of rabbit intestinal coccidiosis: significant distension of the small intestine; many gray-white nodules and numerous hemorrhagic spots on the intestinal wall of the jejunum and ileum; significant thickening of the intestinal wall; and the appearance of the brain in severe cases. Pathological changes in the r*Ei*-14-3-3 and r*Ei*-GRA10 immunized groups were milder: the intestinal wall had a low number of hemorrhagic spots and nodules, and only individual experimental rabbits showed a slight thickening of the intestinal wall (Figure 4). The mean intestinal lesion score for each group was 1.27 for both the r*Ei*-14-3-3 and r*Ei*-GRA10 immunized groups, which was significantly different from that of the PBS-infected group (2.3) and Trx-His-S-infected group (2.6) (*p* < 0.05).

No *E. intestinal* oocysts were found in the stool samples of the blank control group; the mean OPG reached 2.13 × 10^5^ in the PBS-infected group and 1.72 × 10^5^ in the Trx-His-S-infected group, while the mean OPG was significantly reduced in both the r*Ei*-14-3-3 and r*Ei*-GRA10 immunized groups compared to the attacked control group, at 2.95 × 10^4^ (*p* < 0.05) and 3.22 × 10^4^ (*p* < 0.05), respectively. The decreased proportions of oocyst reached 86.13% and 84.87% in the r*Ei*-14-3-3 and r*Ei*-GRA10 immunized groups, respectively (Table 1).

The average body weight gain of both the r*Ei*-14-3-3 and r*Ei*-GRA10 immunized groups was significantly higher than that of the PBS-infected group and Trx-His-S-infected group (*p* < 0.05). The relative body weight gain rates of the r*Ei*-14-3-3 and r*Ei*-GRA10 immunized groups reached 81.94% and 73.61%, respectively (Table 1).

### 2.5. Calculation of ACI Value

The ACI values of the r*Ei*-14-3-3 and r*Ei*-GRA10 groups reached 168.24 and 159.91, indicating that they provided good and moderate immune protection against *E. intestinalis* in the rabbits, respectively.

### 2.6. Specific Antibody Levels Detection

**Figure 5:**
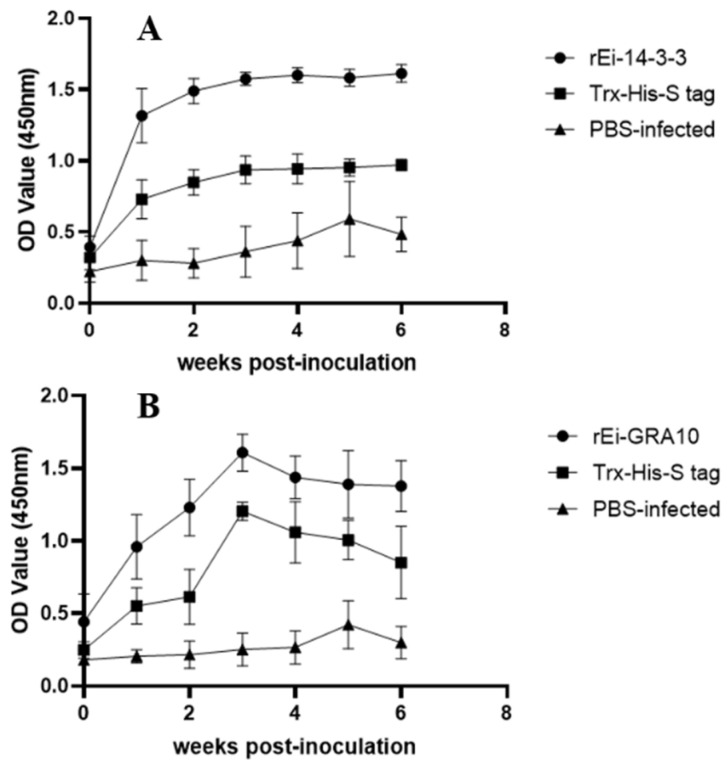
Changes in anti-r*Ei*-14-3-3 (**A**) and anti-r*Ei*-GRA10 (**B**) IgG levels in the sera of each group.

The specific IgG antibody levels of the r*Ei*-14-3-3 and r*Ei*-GRA10 immunized groups increased rapidly after the first immunization and were significantly higher than those in the PBS-infected group (*p* < 0.05). The IgG antibody levels in both immunized groups reached the highest level after the second immunization. The r*Ei*-14-3-3 immunized group maintained a high level after reaching a peak, while the r*Ei*-GRA10 group decreased slightly after reaching the peak, but maintained a high level for 2 weeks after the challenge (Figure 5).

### 2.7. Cytokine Detection

**Figure 6:**
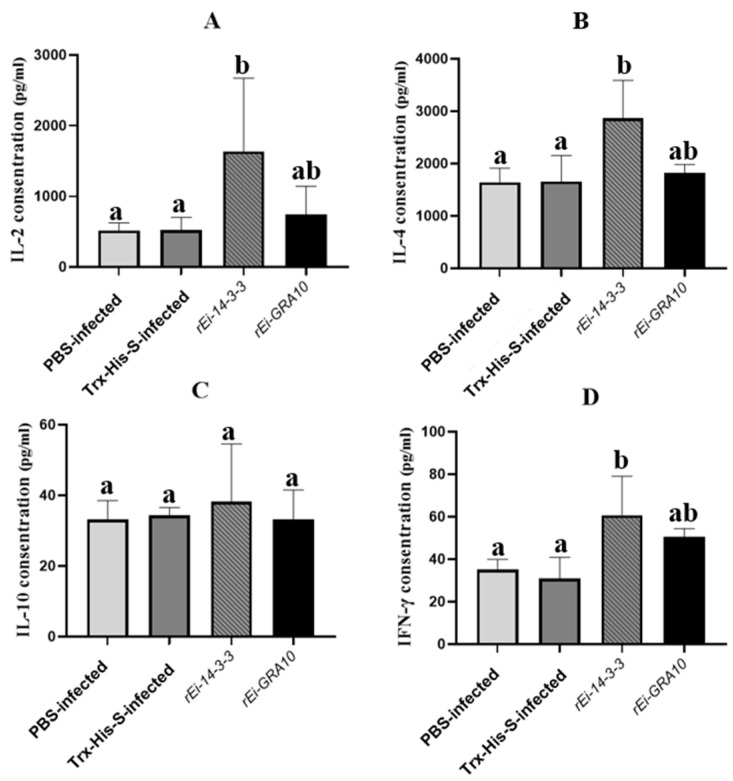
IL-2 (**A**), IL-4 (**B**), IL-10 (**C**), and IFN-γ (**D**) levels in the sera of each group. Different superscripts (a, b) indicate a significant difference (*p* < 0.05). The same superscript indicates no significant difference (*p* > 0.05). The concentration unit of cytokines was pg/mL.

The IL-2, IL-4, and IFN-γ levels were significantly higher in the r*Ei*-14-3-3 immunized group than in the control groups (*p* < 0.05). Although the IL-2 and IFN-γ levels in the r*Ei*-GRA10 immunized group were higher than in the control groups, the difference was not significant (*p* > 0.05) (Figure 6).

## 3. Discussion

Though still in its early stages, research on recombinant subunit vaccines for rabbit coccidia appears to have made some strides in recent years. The immunization of rabbits with recombinant *E. stiedae* surface antigen r*Es*-SAG13 [27], apical membrane antigen r*Es*-AMA1 [28], and immune-mapped protein r*Es*-IMP1 [28] resulted in 82.8%, 74.6%, and 80.0% oocyst reductions, respectively, and significantly higher relative body weight growth rates compared to the control group, reaching 88.77%, 78.1%, and 94.4%, respectively. The recombinant *E. magna* surface antigens r*Em*-SAG10 and r*Em*-SAG11 [29], the microneme proteins r*Em*-MIC2 and r*Em*-MIC3 [30], the gametophyte antigen r*Em*-GAM56 [31], and the rhoptry protein r*Em*-ROP17 [31] provided good immune protection after the immunization of the rabbits. There was only one study of a recombinant subunit vaccine against *E. intestinalis*, indicating that r*Ei*-AMA1 and r*Ei*-IMP1 provided moderate immune protection in rabbits, with ACI values of 152.09 and 147.17, respectively [17].

The 14-3-3 gene contributes to the invasion and growth of coccidia [20,21], and nucleic acid vaccines comprising the 14-3-3 gene have been proven to provide good immune protection against coccidiosis in chickens [32]. In our experiment, the r*Ei*-14-3-3 immunized group achieved 86.13% oocyst reduction, 81.94% relative weight gain, and an ACI value of 168.24, indicating a good immunoprotective effect. In a previous study [32], after immunizing chickens with a DNA vaccine carrying the 14-3-3 antigen gene of *E. hepatica*, the vaccine induced cross-protection against *E. hepatica*, *E. tenella,* and *E. maxima* infections, with oocyst reduction rates of 77.05%, 72.04%, and 58.79% and ACI values of 171.31, 178.29, and 161.03, respectively. This result suggests that the 14-3-3 protein might provide effective protection against infections by different species of coccidia as a common antigen [32]. In our study, the amino acid sequence similarity between three of the rabbit coccidia pathogens (*E. intestinalis*, *E. stiedae,* and *E. magna*) exceeded 99%, suggesting that they are highly conserved. Therefore, r*Ei*-14-3-3 might be able to produce cross-protective effects against other rabbit coccidias.

The dense granule protein family is necessary for Apicomplexan parasites to grow and develop within host cells [22]. Several GRAs of *Toxoplasma gondii* (*Tg*-GRAs) including *Tg*-GRA1, 2, 3, 4, 5, 6, 7, 8, 10, 14, 15, 24, and 41 are effective protective antigens [26]. In a previous study [33], mice were immunized with a multiple antigenic peptide (MAP) vaccine carrying three dominant antigenic epitopes of *Tg*-GRA10. The vaccine immunization activated Th1-type immune responses, with significantly higher IFN-γ, IL-2, and specific antibody IgG levels, as well as higher proportions of CD4+ and CD8+ cells compared with those in the controls, and significantly extended the survival time of mice that were acutely infected with *T. gondii*, as well as decreased the cyst burden in mice with chronic infection [33]. GRA4, 14, and 17 of *Neospora caninum* have also been reported to be immunoprotective [34]. For the coccidia, after the immunization of chickens with the recombinant protein of *Et* (*Eimeria tenlla*)-GRA12, the immunized group showed a notably higher survival rate (100%) than the control group (40%), a higher relative body weight growth rate (38%) than the control group (−46%), a significantly lower mean cecum lesion than the control group, and an oocyst reduction rate of 29.95% [35], indicating a certain immunoprotective effect. In our experiments, the r*Ei*-GRA10 immunized group showed a relative weight gain of 73.61%; an oocyst reduction of 84.87%; and an ACI value of 159.91 with moderate immunoprotection, indicating that GRA10 might be a vaccine candidate antigen in the dense granule protein gene family of coccidia.

In this study, both immunized groups showed notably higher specific IgG antibody levels than the control groups, and both remained at high levels 2 weeks after the challenge, indicating that both r*Ei*-14-3-3 and r*Ei*-GRA10 induced significant humoral immune responses in rabbits.

IL-2 and IFN-γ, which are critical cytokines of the Th1-type immune response, can modulate the immune response of T cells against invasion by pathogenic microorganisms such as coccidia [36,37]. IFN-γ enhances the function of macrophages, natural killer (NK) cells, and cytotoxic T lymphocytes [38], while IL-2 promotes the differentiation and appreciation of cytotoxic T lymphocytes [39]. It has been suggested that the reduction in IL-2 and IFN-γ might lead to the weakening of chickens’ resistance to coccidia [40]. IL-4, a characteristic cytokine of the Th2-type immune response, activates B cells and plays an essential role in the regulation of humoral immunity [41,42]. In our study, r*Ei*-14-3-3 activated the Th1-type immune response in rabbits, as evidenced by the significantly higher IL-2 and IFN-γ levels in the r*Ei*-14-3-3 immunized group (*p* < 0.05) compared with those in the control groups. Furthermore, r*Ei*-14-3-3 also activated the Th2-type immune response in rabbits, as evidenced by significantly higher levels of IL-4 (*p* < 0.05).

## 4. Materials and Methods

### 4.1. Animals, Parasites and Sera

Sera from rabbits infected with *E. intestinalis* (positive) and uninfected rabbits (negative), as well as unsporulated oocysts, sporulated oocysts, merozoites, and gametophytes of *E. intestinalis*, were supplied by the Department of Parasitology, Sichuan Agricultural University.

Fifty 30-day-old coccidian-free New Zealand rabbits were raised in our laboratory. Rabbit cages, troughs, and kettles were regularly flame-sterilized, and the feed was dried at a high temperature. Anticoccidial drugs were added to the drinking water before challenge [27]. From 1 week before challenge, feeds without anticoccidial medications were added.

### 4.2. Synthesis of cDNA

Total RNAs were isolated from the four stages of *E. intestinalis* using an RNA Extraction Kit (Tiangen, Beijing, China). The extracted RNA was used as a template to synthesize cDNA using a reverse transcription kit (Thermo Fisher Scientific, Waltham, MA, USA).

### 4.3. Bioinformatic Analysis

The open reading frame (ORF) and amino acid sequence were obtained using NCBI ORF Finder (https://www.ncbi.nlm.nih.gov/orffinder/, accessed on 16 March 2023). Signal peptides and transmembrane regions were predicted using TMHMM Server v.2.0 (https://services.healthtech.dtu.dk/services/TMHMM-2.0/, accessed on 16 March 2023) and SignalP5.0 (https://www.novopro.cn/tools/signalp.html, accessed on 16 March 2023). The molecular weight (MW) and isoelectric point (pI) were predicted using ExPASy—ProtParam tool (https://web.expasy.org/protparam/, accessed on 16 March 2023). B-cell epitopes were predicted using the analysis resource of the Immune Epitope Database (IEDB) (http://tools.immuneepitope.org/bcell/, accessed on 16 March 2023). Secondary structure prediction and sequence similarity analysis were carried out using Jalview 2.11.2.0 [43].

### 4.4. Cloning of the Ei-14-3-3 and Ei-GRA10 Genes

Primer Premier 5 (Premier Biosoft, Palo, Alto, CA, USA) was used to design the specific primers for *Ei*-14-3-3 and *Ei*-GRA10 based on the transcriptome data. Restriction enzyme sites were added upstream and downstream, respectively, of the primers (Takara, Dalian, China) (Table 2). 

The *Ei*-14-3-3 and *Ei*-GRA10 genes were amplified via PCR using the synthesized cDNA as the template. The PCR products were transformed into *E. coli* DH5α competent cells (Tiangen) after ligation into the pMD-19 T vector (Takara), and the products were subsequently sent for sequencing to determine the correct constructs (Sangon Biotech Co, Shanghai, China).

### 4.5. Expression and Purification of Recombinant Proteins

The constructed pMD19-T-*Ei*-14-3-3 and pMD19-T-*Ei*-GRA10 plasmids were double-enzyme digested and then subcloned into the pET-32a (+) vector (Tiangen). The recombinant pet-32a (+)-*Ei*-14-3-3 and pET-32a (+)-*Ei-*GRA10 plasmids were transformed into *E. coli* BL21 (DE3) (Tiangen). Expression of the recombinant proteins was then induced using 1 mmol/L Isopropyl β-D-1-Thiogalactopyranoside (IPTG). The recombinant proteins were purified using His-Trap HP columns (Cytiva, Marlborough, MA, USA). The results of expression and purification were validated using SDS-PAGE. The purified pET-32a empty protein (Trx-His-S tag), applied as a control, was stored in our laboratory at −80 °C.

### 4.6. Western Blotting

The SDS-PAGE-separated r*Ei*-14-3-3 and r*Ei-*GRA10 proteins were transferred onto polyvinylidene fluoride (PVDF) membranes (Boster, Wuhan, China). The *E. intestinal* positive/negative rabbit serum (diluted at 1:200) antibody and the horseradish peroxidase (HRP)-labeled goat anti-rabbit IgG (Boster) (diluted at 1:1000) were used as the primary and secondary antibodies for immunoblotting analysis of recombinant proteins, respectively.

### 4.7. Immunization Procedure and Experimental Grouping

Fifty 30-day-old coccidia-free rabbits were selected and randomly divided into five groups as shown in Table 3. Subcutaneous neck injections were used to immunize the animals, with the saponin derivative Quil-A being used as the immunoadjuvant. The immunization doses of each group are shown in Table 3.

**Figure 7:**
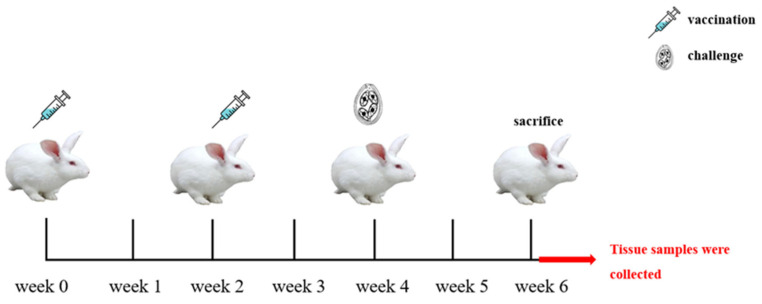
The immunization program.

The first immunization was given to the rabbits at 37 days of age and the second was given at the same dose 2 weeks later. Except for the uninfected group, each experimental rabbit was orally inoculated with 7 × 10^4^ sporulated oocysts at 2 weeks after the second immunization, and all rabbits were sacrificed at 2 weeks after challenge (Figure 7). From before the first immunization (week 0) to the sacrifice (week 7), the sera were collected regularly every week and stored at −20 °C.

### 4.8. Evaluation of the Protective Effect

The protective effect of the recombinant protein was evaluated according to clinical signs, relative weight gain rate (%), oocyst decrease rate (%), mean lesion score, and anticoccidial index (ACI) of animals of each group [44].

Weight gain: The weight of each experimental rabbit was recorded regularly on a weekly basis from before the first immunization (week 0) to the sacrifice (week 7). Mean weight gain (ΔW1) = weight before challenge − weight before first immunization; (ΔW2) = weight before sacrifice − weight before challenge; relative weight gain rate = (average ΔW2 in each group/average ΔW2 in the PBS-uninfected group) × 100%.

Oocyst decrease rate: Rectal fecal samples were collected from each experimental rabbit after sacrifice, and the numbers of oocysts per gram of feces (OPG) were counted for each group. Oocyst decrease rate = (PBS-infected group OPG − each group OPG)/(PBS-infected group OPG) × 100%.

Mean lesion score: Samples of jejunum and ileum were collected from the experimental rabbits after sacrifice. The intestinal lesions of each experimental rabbit were scored, and the mean lesion score of each group was calculated [45].

ACI = (survival rate + relative weight gain rate) − (lesion value + oocyst value). Survival rate = the number of surviving rabbits/total number of rabbits in this group × 100%; lesion value = mean lesion score of each group × 10; oocyst value was determined by the oocyst reduction rate (oocyst value was 0 for an oocyst reduction rate of more than 99%, 1 for 75–99%, 10 for 50–75%, 20 for 25–50%, and 40 for less than 25%). ACI < 120 means no anticoccidial effect; 120 ≤ ACI < 160 means a moderate anticoccidial effect; 160 ≤ ACI ≤ 180 means a good anticoccidial effect; and ACI > 180 means an excellent anticoccidial effect [46].

### 4.9. Specific IgG Antibody and Cytokine Level Detection

The sera were collected weekly before the first immunization until the sacrifice period. The specific IgG antibody levels in the serum samples were determined using indirect enzyme-linked immunosorbent assay (ELISA). Serum samples were diluted 1:100 and HRP-labeled goat anti-rabbit IgG was used as the secondary antibody.

The cytokines (interferon gamma (IFN-γ), interleukin (IL)-2, IL-4, and IL-10) levels in the sera of the rabbits at 2 weeks after the second immunization were determined using the corresponding cytokine ELISA kits (Solarbio, Beijing, China).

### 4.10. Data Analysis

The differences among groups for body weight gains and specific antibody levels were analyzed using one-way analysis of variance (ANOVA) in IBM SPSS statistics 22.0 (IBM Corp., Armonk, NY, USA). And the differences among groups for the number of oocysts excreted and cytokine levels were analyzed using Kruskal–Wallis nonparametric tests in IBM SPSS statistics 22.0. *p* < 0.05 and *p* < 0.01 represent significant and highly significant differences, respectively.

## 5. Conclusions

In this study, we obtained recombinant *E. intestinalis* 14-3-3 and GRA10 proteins (r*Ei*-14-3-3 and r*Ei*-GRA10) via prokaryotic expression. The results show that both r*Ei*-14-3-3 and r*Ei*-GRA10 significantly ameliorated body weight loss, intestinal lesions, and oocyst excretion in rabbits, with ACI values of 168.44 and 159.91, and with good and moderate immunoprotective effects, respectively. Both r*Ei*-14-3-3 and r*Ei*-GRA10 could induce humoral immunity in rabbits. In addition, r*Ei*-14-3-3 could activate the Th1-type and Th2-type immune responses in rabbits. Comparatively, r*Ei*-14-3-3 provided better immune protection than r*Ei*-GRA10 and is thus an ideal candidate antigen for rabbit coccidia recombinant subunit vaccines.

## Figures and Tables

**Table 1:** Statistical results of each data item.

Groups	Average Body Weight Gain (g)	Relative Body Weight Gain Rate (%)	Oocyst Shedding Per Rabbit (×10^4^/g)	Oocyst Decrease Ratio (%)	Mean Lesion Score	Survival Rate (%)	Anticoccidial Index (ACI)
Uninfected	360.00 ± 39.44 ^a^	100	0 ^a^	—	0 ^a^	100	—
PBS-infected	140.00 ± 61.46 ^b^	38.89	21.26 ± 3.07 ^b^	0	2.3 ^b^	100	—
Trx-His-S tag	170.00 ± 42.16 ^b^	47.22	17.18 ± 1.68 ^b^	19.20	2.6 ^b^	100	81.22
r*Ei*-14-3-3	295.00 ± 55.03 ^c^	81.94	2.95 ± 2.15 ^c^	86.13	1.27 ^c^	100	168.24
r*Ei*-GRA10	265.00 ± 66.87 ^c^	73.61	3.22 ± 1.80 ^c^	84.87	1.27 ^c^	100	159.91

Note: Different letters in the same column indicate significant differences (*p* < 0.05), and the same letters in the same column indicate no significant differences (*p* > 0.05).

**Table 2:** Primers for Ei*-*14-3-3 and Ei*-*GRA10.

Genes	Primers	Primer Sequences
14-3-3	Upstream	5′-CGGGATCCATGATTGAGGATATAAAGACCG-3′ (Bam HI site underlined)
	Downstream	5′-CCCTCGAGCTACTGCTGCTCAGTAGCCT-3′ (Xho I site underlined)
GRA10	Upstream	5′-CGGGATCCATGCGACATTACGACATGA-3′ (Bam HI site underlined)
	Downstream	5′-CCAAGCTTTTACCCAACCATAGTGTGG-3′ (Hind III site underlined)

Note: The underlined sequences indicate the sequences of restriction enzyme sites.

**Table 3:** Grouping and dosing of the experimental rabbits.

Groups	Number of Rabbits	Immunogen and Dosage	Challenge
Uninfected	10	1 mL of Sterile PBS	—
PBS-infected	10	1 mL of Sterile PBS	7 × 10^4^ sporulated oocysts
Trx-His-S-infected	10	100 μg Trx-His-S tag + 1 mg Quil-A dilution in 1 mL of PBS	7 × 10^4^ sporulated oocysts
r*Ei*-14-3-3	10	100 μg r*Ei*-14-3-3 + 1 mg Quil-A dilution in 1 mL of PBS	7 × 10^4^ sporulated oocysts
r*Ei*-GRA10	10	100 μg r*Ei*-GRA10 + 1 mg Quil-A dilution in 1 mL of PBS	7 × 10^4^ sporulated oocysts

## Data Availability

All data generated or analyzed during this study are included in this published article.
